# Functional Mapping of Human Dynamin-1-Like GTPase Domain Based on X-ray Structure Analyses

**DOI:** 10.1371/journal.pone.0071835

**Published:** 2013-08-19

**Authors:** Julia Wenger, Eva Klinglmayr, Chris Fröhlich, Clarissa Eibl, Ana Gimeno, Manuel Hessenberger, Sandra Puehringer, Oliver Daumke, Peter Goettig

**Affiliations:** 1 Department of Molecular Biology, University of Salzburg, Salzburg, Austria; 2 Crystallography, Max-Delbrück-Center for Molecular Medicine, Berlin, Germany; 3 Macromolecular Crystallography, Helmholtz-Zentrum Berlin für Materialien und Energie, Berlin, Germany; 4 Department of Biology and Chemistry, Freie Universität Berlin, Berlin, Germany; National Cancer Institute, United States of America

## Abstract

Human dynamin-1-like protein (DNM1L) is a GTP-driven molecular machine that segregates mitochondria and peroxisomes. To obtain insights into its catalytic mechanism, we determined crystal structures of a construct comprising the GTPase domain and the bundle signaling element (BSE) in the nucleotide-free and GTP-analogue-bound states. The GTPase domain of DNM1L is structurally related to that of dynamin and binds the nucleotide 5′-Guanylyl-imidodiphosphate (GMP-PNP) via five highly conserved motifs, whereas the BSE folds into a pocket at the opposite side. Based on these structures, the GTPase center was systematically mapped by alanine mutagenesis and kinetic measurements. Thus, residues essential for the GTPase reaction were characterized, among them Lys38, Ser39 and Ser40 in the phosphate binding loop, Thr59 from switch I, Asp146 and Gly149 from switch II, Lys216 and Asp218 in the G4 element, as well as Asn246 in the G5 element. Also, mutated Glu81 and Glu82 in the unique 16-residue insertion of DNM1L influence the activity significantly. Mutations of Gln34, Ser35, and Asp190 in the predicted assembly interface interfered with dimerization of the GTPase domain induced by a transition state analogue and led to a loss of the lipid-stimulated GTPase activity. Our data point to related catalytic mechanisms of DNM1L and dynamin involving dimerization of their GTPase domains.

## Introduction

Members of the dynamin superfamily comprise a family of conserved GTPases, which are mostly found in the eukaryotic kingdom and mediate functions typically related to membrane remodeling [1], [2]. A defining feature of dynamin superfamily members is a large GTPase domain of roughly 300 amino acids, which distinguishes it from other signaling GTPases. Despite variations in size, the GTPase domains of most dynamin superfamily members contain five conserved GTP-binding motifs (G1-5), similar to small Ras-like GTPases [3]. The P-Loop (G1) or GXXXXGKS/T motif is also present in ATPases (Walker A motif) and functions as a coordinator of the phosphate groups of the bound nucleotide [4]. A conserved threonine in switch-I (G2) and the conserved residues DxxG of switch-II (G3) are involved in Mg^2+^ binding and GTP (Guanosine-5′-triphosphate) hydrolysis. These regions are rather flexible in the GDP-bound form but are stabilized in GTP-bound state [5]. The nucleotide binding affinity of dynamins is typically low, with specificity for GTP provided by the mostly conserved N/TKxD motif (G4) [2], [3]. The G5-motif is involved in binding the ribose moiety.

Although dynamins display a rather high basal GTP turnover rate, additional stimulation (10–100-fold) has been observed for some superfamily members due to self-assembly and lipid-binding [2], [6]–[8]. GTP hydrolysis results in conformational changes that might be necessary for their function as mechanochemical enzymes [8]–[12].

Besides the GTPase domain, dynamin superfamily members share at least two more characteristic sequences: a middle domain and a C-terminal GTPase effector domain (GED) [1], [2]. These sequences constitute two distinct domains, the stalk and the bundle signaling element (BSE). The latter comprises three helices located at the N- and C-terminus of the GTPase domain and at the C-terminus of the GED, respectively [8], [13]. The BSE was proposed to mediate nucleotide-dependent conformational changes from the GTPase domain to the stalk and to regulate dynamin activity in membrane fission [12], [14]. The middle domain and the amino-terminal portion of the GED form an antiparallel four-helix bundle, the stalk of dynamin superfamily proteins [15]–[17]. This stalk mediates dimerization and tetramerization, and the formation of higher-ordered structures, such as rings or spirals [18]–[20]. Lipid binding motifs, such as the PH domain in dynamin or the lipid-binding loop L4 in MxA, are situated at the tip of the stalk, at the opposite end of the GTPase domain [20].

Despite their shared structural and biochemical properties, dynamin superfamily members possess distinct differences in their domain architecture related to their diverse cellular functions [1]. For example, human dynamin-1-like protein (DNM1L, formerly dynamin-related protein 1, Drp1) and optic atrophy 1 (OPA1) have essential roles in controlling mitochondrial dynamics. DNM1L is targeted from the cytoplasm to the outer mitochondrial membrane (MOM) and is a key player in mitochondrial fission [21]. Similarly, DNM1L has a major role in the segregation of peroxisomes [22]. While DNM1L possesses the typical features of the dynamin superfamily, its domain organization includes a sequence insertion of 80 to 130 amino acids between the middle domain and GED. This so-called insert B, or variable domain (VD), displays low sequence conservation and its function has yet to be elucidated in detail. Recently, it has been proposed that alternative splicing and posttranslational modifications within the VD indirectly regulate enzyme activity [23]. Such modifications include phosphorylation, ubiquitination, SUMOylation and S-nitrosylation. The exact mechanisms and effects of these posttranslational modifications on DNM1L activity are controversial. However, it is apparent that tight regulation of DNM1L is necessary to ensure proper mitochondrial function, as abnormal DNM1L activity is associated with excessive mitochondrial fission in various neurodegenerative diseases. Therefore, DNM1L is considered as a potential therapeutic target [24]–[26].

The proposed mechanism for DNM1L in mitochondrial fission shows similarities to the role of dynamin-1 in endocytosis [1]. Similar to dynamin-1, DNM1L exists in a dimer-tetramer-equilibrium in solution [27], [28]. Intracellular cues direct DNM1L to the mitochondrial outer membrane (MOM) where it forms higher-ordered oligomers, which are visible as punctate structures [29]. Interestingly, some DNM1L clusters are located at future mitochondrial scission sites, which are hallmarked by endoplasmic reticulum (ER) contacts [30]. Although a known lipid-binding domain, like the dynamin-1 PH-domain, is missing, *in vitro* studies showed that DNM1L and the yeast homologue DNM1 bind to negatively charged lipids [1], [31]–[33]. Membrane-anchored proteins, such as the mitochondrial fission factor 1 (Fis1) and the membrane fission factor (MFF), have been proposed to mediate DNM1L-membrane binding [34], [35]. Further studies, mainly with yeast DNM1, revealed that the addition of a non-hydrolyzable GTP analog triggers DNM1 self-assembly, while an excess of hydrolyzable GTP causes dissociation of the complex [36]. These findings suggest that GTP binding, but not GTP hydrolysis, is necessary for lipid-free DNM1L assembly. Electron microscopy studies demonstrated that nucleotide-free yeast DNM1 and human DNM1L form large helical spirals with a diameter of ∼120 nm around lipid tubes [32], [33]. Constriction of these spirals to smaller diameters and dissociation from the lipid layer was observed in the presence of GTP. It was therefore suggested that GTP hydrolysis is the driving force for intra-molecular rearrangements, which are necessary for mitochondrial fission events [32]. Comparison of the cryo-EM structures of DNM1 in the constricted and non-constricted forms revealed differences to dynamin-1 assembly and constriction mechanisms [32], [33].

In the current manuscript, we report the crystal structures of human DNM1L GTPase-BSE fusion protein in the nucleotide-free form and in the presence of a non-hydrolyzable GTP analogue, 5′-Guanylyl imidodiphosphate (GMP-PNP). The structural comparison of DNM1L with other dynamin superfamily members led to the identification of highly conserved active site residues, which in dynamin-1 and *A. thaliana* Drp1A are required for the basal and liposome-stimulated GTPase reaction [8], [37]. These residues were systematically mutated to alanine in DNM1L and the resulting mutants kinetically characterized. In addition, residues involved in GTPase domain dimerization and in the unique 80-loop were mutated and functionally analyzed. Based on our detailed structure-function map of the DNM1L active site, we postulate a common mechanism in the GTPase reaction of DNM1L and dynamin-1.

## Materials and Methods

### Cloning and Protein Purification

Cloning, expression and purification of the human DNM1L GTPase-GED (GG) fusion construct was performed as described previously [38]. It starts with the N-terminal Met1 and terminates with an artificial LEHHHHHH-tag at the C-terminal Trp736, corresponding to the numbering of DNM1L isoform 1. Several mutations were introduced into full-length DNM1L isoform 2 in pET21 (GenBank Accession Number NM_012063.2) either by overlap PCR (Q34A, S35A, K38A, S39A, S40A, E81A, E81A/E82A, D146A, D190A, K216A, D218A, N246A) or round the horn cloning (T59A, G149A). Expression and purification of DNM1L full-length and mutants was performed as described elsewhere [39].

### Protein Crystallization, Data Collection and Processing

Nucleotide-free protein crystals were obtained after 3–5 days in a buffer containing 0.1 M sodium citrate pH 5, 27.5% PEG 3000 as described [38]. 1 µl reservoir solution was mixed with 1 µl of protein solution (1.2 mg ml^−1^) and equilibrated against a reservoir volume of 400 µl in a 24-well plate (Hampton Research). Co-crystallization of DNM1L with a non-hydrolyzable GTP analog was attempted with protein samples that were incubated with 1 mM GMP-PNP (5′-Guanylyl imidodiphosphate hydrate) and 4 mM MgCl_2_, and then purified by gel filtration. However, this procedure led to the dissociation of the nucleotide (see results section on determinants of GTPase domain dimerization). Correspondingly, protein crystals grew under the same conditions as nucleotide-free protein crystals and were of similar shape and size. In order to ensure nucleotide binding of the DNM1L GG fusion protein, crystals were soaked supplementary for 2 min by adding solid GMP-PNP powder to the crystallization drop. Both nucleotide-free and nucleotide-bound protein crystals were flash-cooled in liquid nitrogen without additional cryo-protectant. Diffraction data were collected on beamline BL14.1 operated by the Helmholtz-Zentrum Berlin (HZB) at the synchrotron BESSY II (Berlin-Adlershof, Germany) [40]. Diffraction images were recorded at a wavelength of 0.91814 Å using a Rayonics MX-225 3×3 CCD detector. Both data sets were integrated with iMosflm 1.0.7 and scaled with SCALA 3.3.20 for orthorhombic crystals of space group P2_1_2_1_2 with cell constants around 53 Å (a), 151 Å (b), and 43 Å (c) for a maximum resolution of 2.3 Å [41], [42]. See Table 1 for detailed values.

**Table 1:** Data collection and processing statistics.

Data collection	DNM1L nucleotide-free	DNM1L-GMP-PNP
Space group	P2_1_2_1_2	P2_1_2_1_2
Unit cell parameters (Å)	a = 53.50 b = 151.43 c = 42.76	a = 53.42 b = 151.31 c = 43.06
	α = β = γ = 90.0°	α = β = γ = 90.0°
Wavelength (Å)	0.91841	0.91841
Resolution (Å) (highest shell)	75.72–2.30 (2.42–2.30)	37.83–2.30 (2.42–2.30)
Reflections observed*	54279 (7783)	65938 (6133)
Unique reflections*	15371 (2204)	15894 (2027)
Multiplicity*	3.5 (3.5)	4.1 (3.0)
Completeness of data (%)*	96.6 (96.8)	98.1 (88.8)
aR_merge_ (%)*	11.8 (44.9)	12.8 (53.7)
bR_.meas_ (%)*	13.9 (53.2)	14.7 (64.9)
cR_pi.m_ (%)*	7.2 (27.8)	7.0 (35.5)
dI/σ (I)*	7.9 (2.6)	8.4 (2.1)
Protein molecules per asymmetric unit	1	1
Solvent content (%)	41.8	42.1

Both crystals belong to the orthorhombic space group P2_1_2_1_2 with two 2-fold screw axes (a, b) and a 2-fold axis (c), all angles being 90°. X-ray diffraction reflections were observed with a redundancy (multiplicity) in the range of 3.5 to 4.1, which improved the data quality by averaging observations. In both cases, the overall signal-to-noise ratio I/σ was around 8, reaching its limit of around 2 at the maximum resolution of 2.3 Ångström. The redundancy-dependent factor R_merge_, the redundancy-independent factor R_meas_ (R_rim_), and the precision indicating R_pim_ were calculated as deviations from averaged reflection intensities (I) according to the given formulas and indicate the data quality. Solvent content refers to the volume of disordered aqueous buffer within the protein crystal lattice.

* alues in parentheses refer to the highest resolution shell.

a The redundancy dependent merging R-factor: 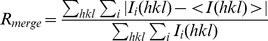.

b The redundancy independent R-factor: 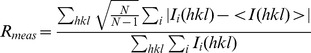.

c The precision indicating merging R-factor: 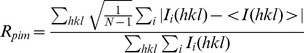.

d Mean (I/sd(I)) from SCALA.

### Phasing and Refinement

Molecular replacement searches were performed with PHASER (version 2.3.0) by using the coordinates of human dynamin-1 (PDB code 3SNH), comprising only the GTPase and BSE domain [43]. A solution with one molecule in the asymmetric unit was obtained for the GMP-PNP complex with a log-likelihood gain (LLG) of +181 and Z values for the rotation function (RFZ) of 7.7 and of 16.0 for the translation function (TFZ). For the nucleotide-free structure, a largely refined polypeptide taken from the GMP-PNP complex was employed to yield LLG = +2789, RFZ = 25.8, and TFZ = 41.5, with an initial R-factor of 43.1. Model building and refinement were done alternately and iteratively with COOT 0.6.2 and PHENIX 1.8–1069, starting with simulated annealing and inspection of composite omit maps calculated in CCP4i, in order to minimize bias from the search model, in particular for the nucleotide-free data, which had a different test set for calculation of R_free_ [44]. This procedure was followed by coordinate and B-factor refinement, water picking, as well as occupancy refinement of disordered loops and side chains in early cycles [45], [46]. In both models, the defined 2F_o_-F_c_ electron density starts with Met1, while some flexible side chains at the surface and, in particular, several loop stretches are not visible. The backbone of switch I starting with Gly54 is largely traceable but the side chains are not well defined until Thr59. Little density is observed from Thr79 to Gly84 in the unique DNM1L 80-loop, as well as for the stretch from Ile118 to Lys123. For Pro325 and the following four residues of the artificial linker not much density is observed. Finally, the last visible C-terminal residue was Thr733. Both ligands, citrate (FLC) and GMP-PNP (GNP) are well resolved. Analyses of the models were performed with programs from the CCP4 package, such as BAVERAGE, MOLPROBITY, and SUPERPOSE (SSM) [44], [47]–[49]. Refinement statistics and quality parameters are summarized in Table 2. Figures of structural models were created with PyMOL, including the electrostatic potential calculation with the APBS plugin using a dielectric constant of 80.0 for water as solvent in the range ±120 k_B_T/e [50]. Coordinates and structure factors of the nucleotide-free and the GMP-PNP bound form were deposited in the RCSB Protein Data Bank under accession codes 4H1U and 4H1V, respectively.

**Table 2:** Refinement and model quality statistics.

Refinement	DNM1L nucleotide-free	DNM1L-GMP-PNP
Resolution (Å) (highest shell)	20.0–2.30 (2.38–2.30)	20.0–2.30 (2.38–2.30)
Reflections*	15331 (1517)	15820 (1322)
Completeness (%)*	95.2 (96.0)	97.8 (84.6)
Working set*	14559 (1451)	15034 (1256)
Test set (5%)*	772 (66)	786 (66)
^a^R_cryst_ (%)*	23.0 (30.9)	22.7 (26.7)
bR_free_ (%)*	26.9 (34.2)	27.8 (31.7)
^c^RMSD bond lengths (Å)	0.005	0.010
^c^RMSD bond angles (°)	0.976	1.097
Number of non-hydrogen protein atoms	2766	2766
Number of ligand atoms	13 (citrate = FLC)	32 (GMP-PNP = GNP)
Number of solvent molecules	129	122
Overall B value (Å^2^)	24.4	43.9
Overall B-factor for protein atoms (Å^2^)	24.7	44.1
Overall B for ligand (Å^2^)	33.6	49.7
Overall B for solvent atoms (Å^2^)	17.8	39.1
**Ramachandran plot**		
Favored regions	340 (95.5%)	338 (94.9%)
Additionally allowed regions	15 (4.2%)	18 (5.1%)
Disallowed regions	1 (0.3%)	0 (0.0%)
**PDB accession codes**	**4H1U**	**4H1V**

The quality indicating factor R_cryst_ was calculated for observed structure factor amplitudes F_obs_ (square root of I) and their model counterparts F_calc_. As independent criterion for the agreement of model atomic coordinates with X-ray data, 5% of reflections were not used for refinement, but for calculation of R_free_. B-factor values that reflect thermal atom motions are in the normal range in both structures. In particular, the nucleotide GMP-PNP with a relatively lower average B-factor appears to be more tightly bound than the unspecific ligand citrate. Deviations from standard bond lengths and angles are low, as indicated by the respective RMSD values. Also the dihedral angles of the polypeptide backbone corroborate the good geometry of both models, with only one outlier in the Ramachandran plots.

* alues in parentheses refer to the highest resolution shell.

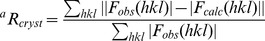
 Rcryst is calculated with 95% of reflections (working set).

b *R_free_* is calculated with the same formula, using 5% of reflections (test set).

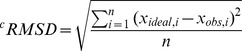
 with x either being bond lengths or angles, for the calculation with ideal and observed parameters. In case of two atomic coordinate sets the average distance of the Cα coordinates from two structures is calculated accordingly.

### Continuous-coupled GTPase Assay

Full-length DNM1L, active site mutants and predicted G-interface dimerization mutants were assayed at a protein concentration of 1.2 µM for their ability to hydrolyze GTP in the range of 0 to 1000 µM based on NADH depletion and absorbance measurement at 340 nm, as described elsewhere [38]. GTPase assays were performed in 25 mM HEPES/PIPES pH 7.0, 150 mM NaCl at 37°C for 84 min and 42 s per cycle. The kinetic parameters represent means of at least three independent measurements. Calculations were performed using GraphPad Prism version 5.0a for Mac OS X, GraphPad Software, La Jolla California USA, (www.graphpad.com). The standard deviation for the k_cat_/K_m_ values was calculated according to Fenner [51].

### GTP Hydrolysis Assays for Lipid Stimulated Activity

Multiple turnover assays in the presence and absence of 0.5 mg/ml phosphatidylserine (PS) liposomes were carried out using 10 µM DNM1L or the indicated mutants in the presence of saturating GTP concentrations (final concentration 1–1.5 mM) in phosphate buffered saline (PBS, pH 7.4), 2.5 mM DTT, 0.5 mM MgCl_2_ at 37°C, as previously established [33]. The protocol for the preparation of liposomes (http://www.endocytosis.org/techniqs/Liposome.html) was adapted to 100% PS (Avanti Polar Lipids). PS liposomes were chosen because DNM1L binds PS liposomes with similar efficiency to liposomes composed of a mitochondrial outer membrane lipid mixture [33], [39], [52]. GTPase reactions were started by addition of the protein. Within 12 minutes, 5 aliquots were taken (after 1, 2, 4, 5, and 10 min), diluted 1∶20 in PBS and snap-frozen in liquid nitrogen. After thawing, aliquots were immediately applied on an Agilent 1260 Infinity LC (Agilent Technologies), equipped with a reversed-phase ODS-2 Hypersil column (Thermo Scientific). The running buffer contained 10 mM Tetra-N-butylammonium bromide, 100 mM potassium phosphate (pH 6.5) and 7.5% acetonitrile. Denatured proteins were adsorbed on a Nucleosil 100 C18 guard column (Knauer); separated GDP and GTP were detected by measuring the absorption at 254 nm and quantified by integration. Initial turnover rates were derived from a linear fit to the data with less than 40% hydrolyzed GTP. For an assessment of the DNM1L nucleotide specificity, the assay was performed with 1 mM ATP, which showed no significant turnover, i.e., less than 3% compared to GTP in the presence of PS liposomes.

### G-interface Dimerization Studies by Analytical Size-exclusion Chromatography

DNM1L GG fusion protein and mutants at a concentration of 60 µM were incubated with or without 2 mM of the indicated guanine nucleotide analogs for 30 minutes at 37°C in a buffer containing 20 mM Tris/HCl pH 8.0, 150 mM NaCl, 2 mM EGTA, 4 mM MgCl_2_ and 1 mM DTT. In order to mimic the transition state, proteins were incubated in the presence of 2 mM GDP or GTP, 20 mM NaF and 2 mM AlCl_3_. Incubated samples were subjected to size- exclusion chromatography (SEC) on a Superdex S 75 HR 10/300 column and compared with molecular weight markers conalbumin and carbonic anhydrase (GE Healthcare).

## Results and Discussion

### Overall Structure of the Human GTPase-GED Fusion Protein

**Figure 1:**
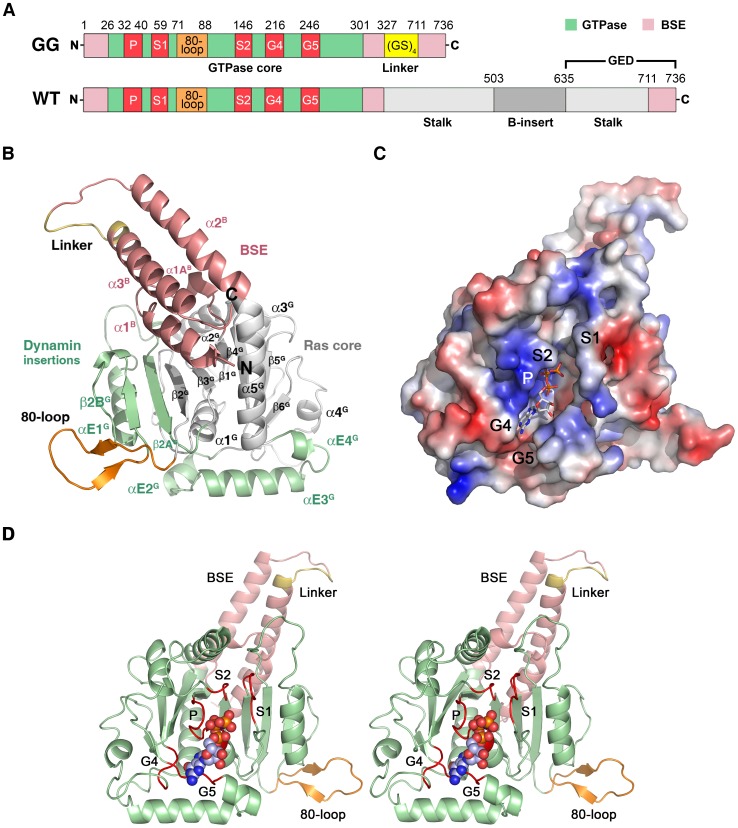
Overall structure of the DNM1L GTPase-GED fusion protein. (**A**) Schematic representation of the construct expressed in *E. coli* and used for crystallization. The GTP binding stretches P-loop, switches I and II (S1 and S2), as well as G4 and G5 are depicted in red. The unique DNM1L insertion, denoted 80-loop, is shown in orange, the artificial (GS)_4_ shortcut in yellow and the bundle signaling element (BSE) in salmon. Also, the GTPase effector domain (GED) is indicated. Residue numbering follows the original human sequence of isoform 1 starting with Met1. The C-terminal linker with His_6_ tag is not included. (**B**) Tertiary structure of the nucleotide-free DNM1L GTPase-GED with secondary structural elements labels. The GTPase core homologous to human Ras is displayed in grey with dynamin-1-typical insertions in green and the BSE in salmon, the shortcut linker in yellow, and the 80-loop in orange. The conformation of the BSE represents the more compact closed or post-fission state of dynamin-like proteins. (**C**) Surface potential representation of the DNM1L GG structure with GMP-PNP shown as stick model bound in the active site cleft, turned around the y-axis by 180° with respect to Fig. 1B. The electrostatic potential at the molecular surface ranges from −120 to +120 k_B_T/e, with negatively charged regions depicted in red and positively charged ones in blue. (**D**) Stereo view of nucleotide-bound DNM1L GTPase-GED. GMP-PNP is depicted as atomic sphere model bound in the active site cleft with the nucleotide binding stretches colored in red. Otherwise, the color scheme is according to Fig. 1B, except for the whole GTPase domain shown in green. Atom colors are carbon in grey, oxygen in red, nitrogen in blue and phosphorus in orange. This nucleotide-free structure corresponds to the closed or post-fission state.

Similar to previous studies for human dynamin and *Arabidopsis thaliana* A (*At*Drp1A) [8], [37], we designed a fusion protein comprising the GTPase domain (G) and the bundle signaling element (BSE, B) of human DNM1L (the so-called GTPase domain - GED (GG) construct [13]). The stalk (S), which participates in higher-order oligomerization, and the B-insert were replaced by a GSGSGSGS linker, which continued with the C-terminal helix of the BSE (Fig. 1A) [38]. This construct was expressed in *E. coli* and purified and crystallized in the absence of nucleotides. To obtain structural insights into nucleotide binding, nucleotide-free crystals were soaked with the non-hydrolyzable GTP analogue GMP-PNP [38]. Both crystals diffracted to 2.3 Å resolution, and the structures were solved by molecular replacement using the GG construct of human dynamin-1 as a search model (Tables 1, 2). Both structures exhibited a monomeric GG construct in the asymmetric unit. All molecular contacts generated by symmetry operations of the space group P2_1_2_1_2 appear to be typical crystal contacts, as indicated by the PISA server (http://www.ebi.ac.uk/msd-srv/prot_int/cgi-bin/piserver). However, a polar interface, generated by the 2-fold axis, might form a functional DNM1L assembly, which is discussed in detail further below.

The GTPase domain of human DNM1L consists of a central eight-stranded β-sheet surrounded by seven α-helices and two one-turn helices resembling the GTPase core of mammalian dynamin-1 [53]. The β-sheet is composed of six parallel and two anti-parallel (ap) strands in the spatial order β6^G^, β5^G^, β4^G^, β1^G^, β3^G^, β2^G^ (ap), β2A^G^ (ap), and β2B^G^. The latter two β-strands represent an insertion including the helix αE1^G^ with respect to the prototypic, canonical GTPase domain of h-Ras (Fig. 1B) [54]. Helices α1^G^ to α5^G^ are present in h-Ras and dynamin-1, while DNM1L shares helices αE2^G^ to αE4^G^ that are inserted between β6^G^ and α5^G^ only with dynamin-1, comprising residues 248 to 281 (Fig. 1B). Although the GTPase domain of DNM1L is similar to that of dynamin-1 at the sequence and structural level, it possesses a unique 16-residue insertion between Ser71 and Glu88, which shall be designated as “80-loop”, according to its central residue (Fig. 1).

The BSE is formed by the N-terminal helices α1^B^ (residues 4–16) and α1A^B^ (20–24), helix α2^B^ (303–323) and the C-terminal helix α3^B^ (708–729, with residues S^708^GS^710^ from the artificial linker), arranged in a three-helix bundle with an additional one-turn helix (Fig. 1B). Both our structures represent the closed conformation of the BSE in relation to the GTPase domain, which has been called the “post-fission state” [37]. Similar closed conformations were found in nucleotide-free and transition state complexes of mammalian dynamin-1 or the GDP bound form of *A. thaliana* Drp1A [3], [8], [16], [17], [37].

### Conformational Changes upon GTP-binding in the Active Site

**Figure 2:**
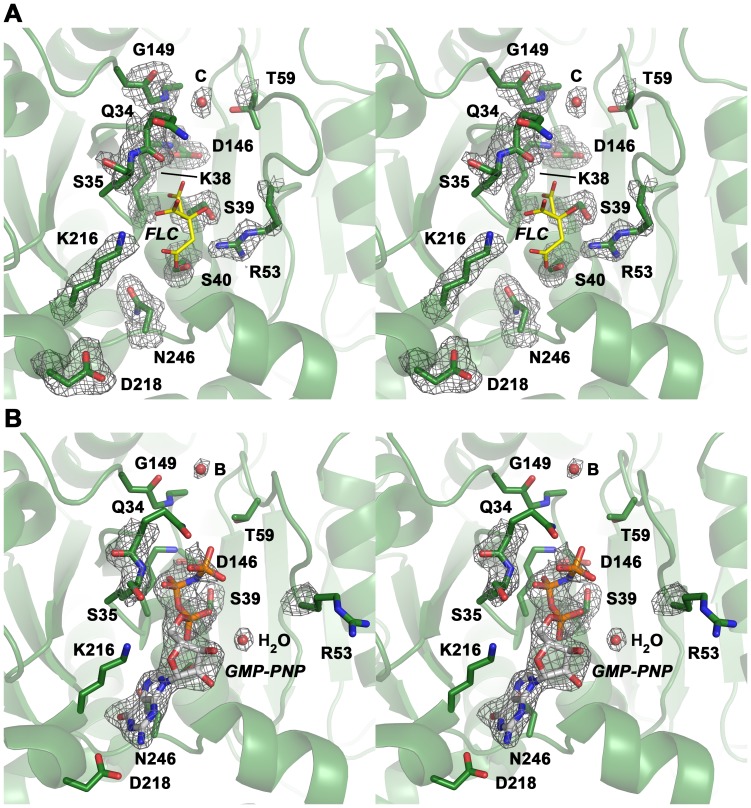
Close-up views of the active site cleft in the nucleotide-free and bound structures of the DNM1L GG construct in stereo. (**A**) The nucleotide-free form with the most relevant residue side chains of the five GTP binding stretches and citrate (FLC, yellow) displayed as stick models. Electron density of a 2F_o_-F_c_ map is shown in grey and contoured at 1σ. The red sphere designates the catalytic water (**C**). (**B**) GMP-PNP complex of the DNM1L GG construct. The nucleotide is shown as stick model, while the red spheres represent water molecules, such as the bridging water (**B**) and one, which binds to the α-phosphate. The electron density of a 2F_o_-F_c_ map is shown in grey and contoured at 1σ, surrounding the nucleotide and relevant parts of the structure with significant conformational changes with respect to the nucleotide-free form.

DNM1L contains the five canonical guanine nucleotide binding motifs: G1 or P-loop (phosphate binding loop) with the highly conserved sequence G^32^SQSSGKSS, switch I with the central T59 as G2 element, G3 around switch II (D^146^LPG) and G4 (T^215^KLD), which are conserved among all GTPases, such as h-Ras. In addition, DNM1L also possesses the dynamin-specific G5 or G-cap motif consisting of G^243^VVNRSQ (Figs. 1C, D) [3]. These motifs surround the active site cleft and exhibit a distinct charge distribution: The positively charged P-loop attracts the anionic triphosphate of GTP, while a less-charged area is complementary to the ribose moiety. The polar part of the guanine base is directed towards the negatively charged G4/G5 region (Fig. 1C). Eventually, the G4 and G5 elements bind guanine, while the P-loop, G4 and G5 bind the ribose moiety and the α to γ-phosphates, and the switch I and II regions mediate γ-phosphate stabilization and hydrolysis. In the nucleotide-free form, a citrate from the crystallization buffer is bound to the P-loop, mimicking the phosphate moiety of GTP (Fig. 2A). Apparently, the citrate compensates charges in the active site of DNM1L, while the GMP-PNP occupied active site exhibits a distinct conformation in the P-loop and switch I (Fig. 2B).

**Figure 3:**
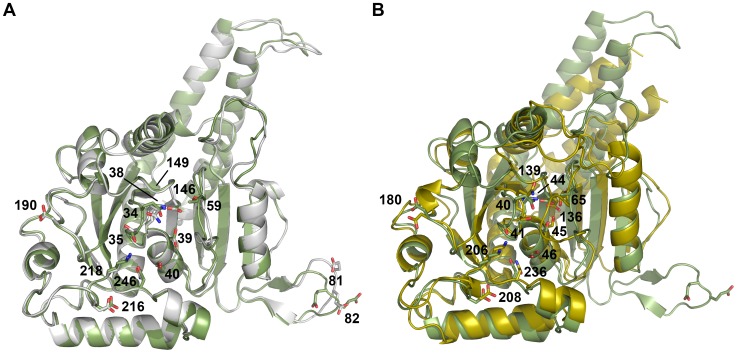
Superposition of the two DNM1L GG structures and dynamin-1 GG. (**A**) Overlay of the nucleotide-free DNM1L GG structure in white with the GMP-PNP-bound structure in green (shown without ligands). Side chains that were mutated in our study are shown as stick models with sequence number labels. (**B**) Overlay of dynamin-1 (PDB code 2X2F) in yellow with the structure of GMP-PNP-bound DNM1L in green. Mutated residues of DNM1L that are equivalent to those of dynamin (see Fig. 3A) are displayed as side chain stick models with dynamin sequence numbers (depicted without ligands).

The overall root mean square deviation (RMSD) between the nucleotide-free and –bound DNM1L GG construct is only 0.69 Å for the C_α_ atoms, indicating almost identical structures (Fig. 3A). The nucleotide-bound form and the corresponding dynamin GDP-AlF_4_^−^ complex (2X2F) exhibit an RMSD value of 2.10 Å (Fig. 3B). However, both DNM1L forms differ significantly in their active site (Figs. 2A, B, and 3A), which shall be discussed in the following.

### The Phosphate Binding Loop (G1)

**Figure 4:**
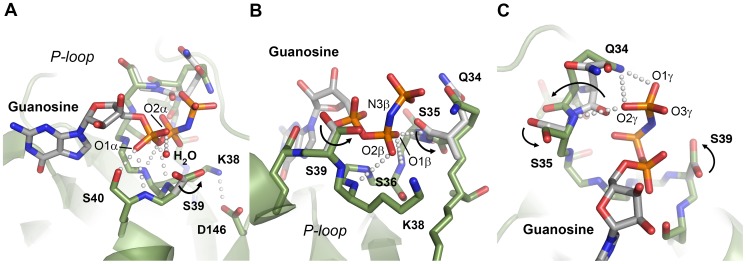
Detailed views of GTP-binding in the P-loop of DNM1L. (**A**) Four hydrogen bonds fix the α-phosphate to the P-loop: O1α to Ser40 Oγ (distance 2.48 Å) and NH (3.45 Å), O3α to NHs of Ser39 (3.34 Å) and Lys38 (3.30 Å). Additionally, the O2α binds an H_2_O (3.39 Å). Lys38 is stabilized by a hydrogen bond to Asp146 from switch II. The overlaid apo-structure in grey shows that the Ser39 side chain rotates about 180° upon GTP binding, to a conformation (in green) that is suitable to stabilize a GTP transition state as seen in other structures of dynamins, e.g. in complex with GDP-AlF_4_^−^. (**B**) Interactions that fix the β-phosphate: O1β forms hydrogen bonds to the NHs of Ser36 (3.31 Å) and of Ser35 (2.94 Å), while the O2β only binds the Lys38 NH (3.49 Å). The Nζ of Lys38 is more than 4 Å away from the O2β, but has the capacity to stabilize together with the Ser39 side chain the phosphate portion of the GTP, as seen in other dynamin-1 nucleotide analogue complexes. (**C**) The γ-phosphate forms hydrogen bonds via its O2γ to the Ser35 NH (3.48 Å) and to the Nε2 of Gln34 (3.14 Å), which also binds the O1γ (2.72 Å). The Gln34 side chain rotates significantly from the apo-conformation (light grey) to the nucleotide-conformation (green). The 180° peptide flip between Gln34-Ser35 brings Ser35 NH in a position suitable for O1β and O2γ binding, accompanied by a 180° side chain rotation of Ser35. Similar peptide flips occur in apo-nucleotide pairs of mammalian and *D. discoideum* dynamins.

The P-loop fixes the triphosphate analogue moiety of GMP-PNP with an intricate hydrogen bonded network (Figs. 4A–C). The α-phosphate is bound by Ser40 via main and side chain interactions, and by backbone interactions of Ser39 and Lys38. Additionally, a single water molecule is coordinated by the α-phosphate (Fig. 4A). The β-phosphate is coordinated by several main chain interactions of Ser35, Ser36, Gly37 and Lys38, which form a tight loop around the β-phosphate. The hydroxyl group of Ser39 rotates about 180° with respect to the nucleotide-free active site. In human dynamin-1 with 5′-Guanylylmethylenediphosphonate (GMP-PCP) (3ZYC) and GDP-AlF_4_^−^ (2X2E), or plant Drp1A with GDP-AlF_4_^−^ (3T34), the side chains of Lys44/47 (corresponding to Lys38 in DNM1L) and Ser45/48 (Ser39 in DNM1L) are involved in β and γ-phosphate binding. In contrast, the side chain of Lys38 of DNM1L is in a similar position in the nucleotide-free and GMP-PNP bound forms and does not bind to the β-phosphates (Fig. 4B). Also, Ser39 in the DNM1L structure does not make significant interactions with the β-phosphate.

Compared to the GMP-PCP-bound crystal structure of dynamin-1 (3ZYC), the γ-phosphate of GMP-PNP in DNM1L is shifted by about 2.5 Å towards the Gln34 side chain. This shift is supported by hydrogen bonds of the Gln34 side chain and the Ser35 main chain with the γ-phosphate. Remarkably, the peptide bond between Gln34-Ser35 flips by 180° from its apo-conformation upon GMP-PNP binding, accompanied by a Ser35 side chain rotation of nearly 180° (Fig. 4C). The same peptide bond flip is observed when the apo and GDP-bound structures of *D. discoideum* dynamin-A are compared (1JX2 and 1JWY), as well as the Ser35 and Ser39 side chain rotations, strongly supporting the idea that DNM1L and dynamin use a similar mechanism for nucleotide binding [3].

Ser41 in dynamin, corresponding to Ser35 in DNM1L, is required for binding a catalytic cation, such as Na^+^, in the presence of a transition state analogue of the GTPase reaction (2X2E) [8]. In our DNM1L structure, we did not find a cation at this position. Also, in the GMP-PCP bound structure of a dynamin-1 GG construct (3ZYC), no cation has been found at this position, since it might only be recruited during GTP hydrolysis. It is possible that the bridging nitrogen atom in GMP-PNP favors an unusual conformation of the γ-phosphate, which shifts about 2.5 Å away from the catalytic water with respect to the transition state analogue GDP-AlF_4_^−^ in dynamin-1. This shift might interfere with cation binding of Ser35 in our structure.

### The Catalytic Switches I and II

**Figure 5:**
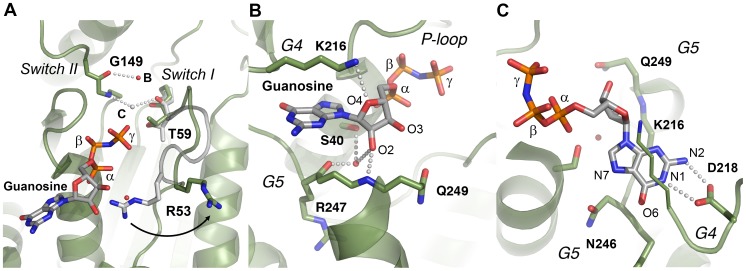
Detailed views of the GTP-binding elements switch I, switch II, G4 and G5 of DNM1L. (**A**) The canonical Mg^2+^ site between O1β and the O2γ is not occupied in the GMP-PNP-DNM1L structure (green). Also, no significant positional shift of Thr59 from the switch I loop takes place between apo- (grey) and nucleotide form (green). The unnatural N3β atom may favour the γ-phosphate conformation rotated by about 60° with respect to the transition state of GTP, shifting it about 2.5 Å away from the catalytic water. Only the nucleotide-free form exhibits the catalytic water molecule (C, grey) bound at the Thr59 carbonyl O (3.15 Å) and connected to switch II via the NH of Gly149 (2.87 Å). The bridging H_2_O (B, red) is only present in the nucleotide complex, bound to the Gly149 carbonyl O (3.21 Å). Upon GMP-PNP binding, the Arg53 side chain moves out of the active site, making room for an H_2_O, which binds O2 of the α-phosphate. (**B**) The ribose of GMP-PNP forms bonds with the ether oxygen O4 to the Nζ of Lys216 (3.16 Å), and with the hydroxyl group of O2 to an H_2_O (3.25 Å), which is bonded to the carbonyl O of Arg247 (2.45 Å) and the Ser40 Oγ (3.01 Å). Another bond is formed by the ribose O2 to Gln249 NH (2.79 Å). (**C**) The Lys216 side chain, depicted as thin stick model for clarity, covers the aromatic rings of the guanine part, while the Asp218 carboxylate binds the amino N2 (2.86 Å) and the N1 (3.12 Å). A further interaction from the Asn246 Oδ1 to the N7 (3.54 Å) might be mediated by an unresolved H_2_O, which could be bound to the carbonyl O of Gly37, as seen in other dynamin-nucleotide complexes.

Interestingly, no Mg^2+^ ion is present between the β- and γ-phosphates in the GMP-PNP-bound DNM1L crystals, as would be expected for a GTP analogue complex. Based on the dynamin-1 GMP-PCP complex (3ZYC), this cation would be coordinated by the side chains of Ser45, Asp146, Thr59, and two water molecules. However, in DNM1L, the C_α_ atom of the central residue from switch I, Thr59, is shifted 2–3 Å away from the canonical Mg^2+^ binding site with respect to corresponding dynamin-GTP analogue complexes (Fig. 5A).

Although the switch I region is not very well defined in the electron density, a large movement of Arg53 is observed (Fig. 5A). In GMP-PNP-DNM1L, the side chain of Arg53 extends to the bulk solvent above the GTP-binding groove, whereas in the nucleotide-free form, this side chain is hydrogen-bonded to Ser40 and Glu43, in a position similar to that of the nucleotide-loaded dynamin complex. This translocation in the nucleotide-free state may be an artificial charge compensation for the negative charge of the P-loop bound citrate.

Based on the crystal structure of the AlF_4_^–^bound GTPase domain dimer of dynamin-1, two water molecules were suggested to directly contribute to catalysis: A catalytic (C) water molecule, positioned near Thr65 in switch I and Gln139 in switch II, is thought to mediate the nucleophile attack on the GTP. A second water molecule orients the catalytic water and additionally bridges Gln40 in the P-loop with Gly139 in switch II (the bridging or B water) [8].

Interestingly, the water molecule in our nucleotide-free DNM1L GG construct is in the same position as the catalytic water in dynamin-1. This catalytic water connects switch I with switch II via hydrogen bonds to the Thr59 carbonyl oxygen and the Gly149 NH, respectively (Figs. 2A and 5A). In contrast, in the nucleotide-loaded DNM1L complex, the carbonyl oxygen of Gly149 binds to a single water molecule, which corresponds to the bridging water molecule in dynamin-1 (Figs. 2B and 5A). Asp146 in switch II is crucial for Mg^2+^ binding in dynamin-1 and *At*Drp1A [8], [37]. It has a similar position in the apo- and GMP-PNP structures but interacts only with the Lys38 side chain (Figs. 2B and 4A).

### Guanosyl Moiety Binding Elements (G4 and G5)

Overall, the rather rigid guanosyl binding pocket of DNM1L with the G4 and G5 elements exhibits more characteristics of a lock-and-key enzyme whereas structural changes in the more flexible P-loop, and the catalytic residues in switch I and II resemble the induced-fit principle. Thus, the P-loop, switch I and switch II in DNM1L undergo major conformational changes upon nucleotide binding, while no conformational changes were observed in G4 and G5. This phenomenon is found in all related dynamin structures, in which G4 and G5 exhibit strikingly similar conformations independent of the nature of the bound nucleotide. The ribose O4 atom is bound by Lys216 in G4, while the 2′ OH group of the ribose is stabilized by water-mediated contacts with the Arg247 main chain in G5 and the Ser40 side chain of the P-loop (Fig. 5B). The guanine moiety of GMP-PNP is located between the G4 and G5 stretches, whereby the Lys216 side chain covers one flat side of the aromatic ring system. Nucleotide specificity is mediated by Asp218, which coordinates the guanine base via two hydrogen bonds (Fig. 5C). Another interaction with the G5 stretch completes the substrate binding elements of DNM1L: Asn246 in G5 forms part of the guanine-binding pocket, and its side chain could interact with N7 of the aromatic ring system via a water molecule, as in dynamin-1 (Fig. 5C).

### The BSE Conformation

**Figure 6:**
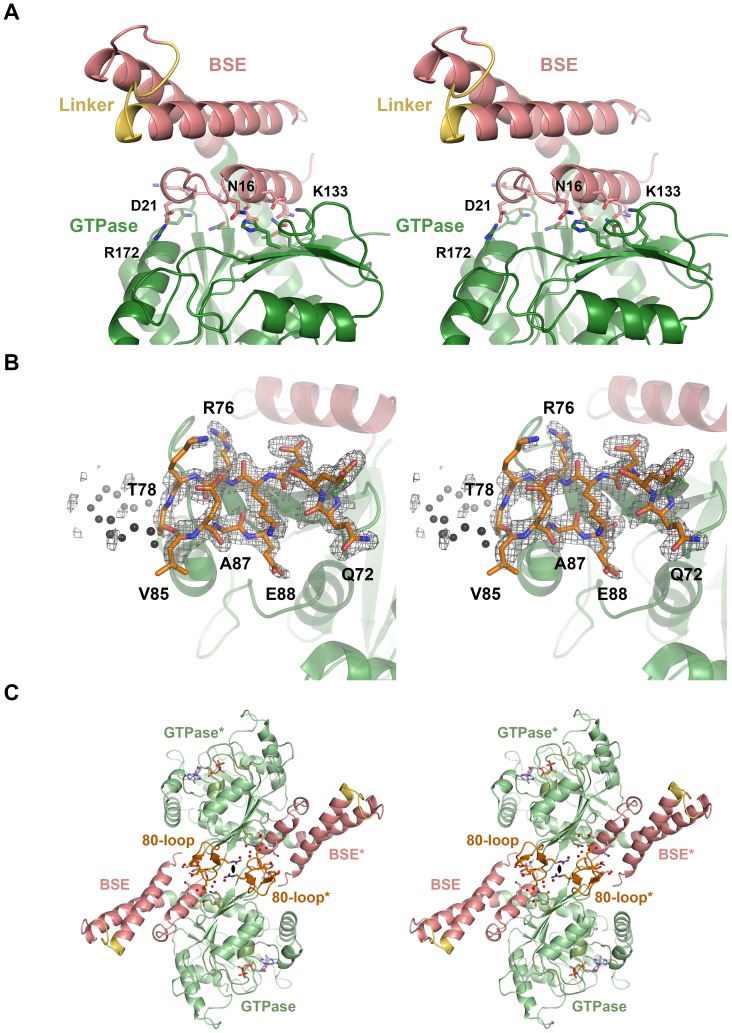
BSE-GTPase domain interface, 80-loop, and their interface at the 2-fold axis. (**A**) The BSE-GTPase interface in the GMP-PNP complex. Both the nucleotide-free and –bound structures represent the closed or post-fission state of dynamin superfamily proteins and exhibit virtually no conformational differences. The interface of GTPase and BSE domain is characterized by mixed charged, polar, and hydrophobic interactions. (**B**) 80-loop insertion in the GTPase domain of DNM1L from Gln72 to Glu87. The insertion exhibits a short antiparallel β-sheet between Arg76 and Ala87. From Thr79 up to Gly84 the electron density of the 2F_o_-F_c_ map, shown in grey and contoured at 1σ, is not well defined. (**C**) Polar interface between two symmetry-related DNM1L monomers A and A*. The 2-fold crystallographic axis (black oval) generates an interface that involves 14 hydrogen bonds and salt bridges of polar and charged side chains from the BSE and the 80-loop, together with roughly 20 water molecules (shown as sticks and red spheres, respectively). Both the GTPase domain and the stalks could form higher oligomers, while this dimer remains intact.

Our nucleotide-free and GMP-PNP DNM1L structures exhibit virtually the same closed or post-fission conformation of the BSE relative to the GTPase domain, with the little defined linker replacing the stalk and B-insert domains (Fig. 1A, B). The interface between the BSE and the GTPase domain is formed by charged, polar, and hydrophobic residues (Fig. 6A). As in the GDP⋅AlF_4_^−^ dynamin-1 and the *At*Drp1A-GDP complex, the connected helices α5^G^ and α2^B^ are kinked at the Leu301-Pro302 bond by about 60°. In dynamin-1, these helices stretch into a single straight helix upon GTP binding, accompanied by a rotation of the complete BSE around the Leu293-Pro294 kink, corresponding to Leu301-Pro302 in DNM1L.

A comparison of the mammalian dynamin-1 apo (pdb code: 3SNH), GDP⋅AlF_4_^_^ (2X2E) and GMP-PCP (3ZYC) forms and the corresponding *At*Drp1 structures (3T34, 3T35) suggests that the mechanochemical energy conversion is accompanied by a twist of the central β-sheet in the GTPase domain, involving a shift of strands β2^G^ and β3^G^ by about 5 Å [8], [12], [16], [17], [37]. The GTP analogue complex does not exhibit the twist of the central β-sheet which might be initiated by GTP-Mg^2+^ binding to the switch I Thr59 and switch II Asp146 together with Gly149. Since our GG construct is tightly packed in the crystals, the GTP hydrolysis transition state and the pre-fission conformation of the BSE are most likely not accessible. Furthermore, conformational changes induced by GTPase domain dimerization might also contribute to the open conformation of the BSE.

### The Unique DNM1L 80-loop

Between the two strands β2^G^ and β2A^G^ of the GTPase domain, DNM1L possesses a unique insertion (residues 72–87), which has only a counterpart in *S. cerevisiae* DNM1 (see section Conclusion). This insertion forms a flexible loop, which is clamped by a short antiparallel β-sheet between Arg76 to Thr78 and Val85 to Ala87 exhibiting an excess of negatively charged side chains (Fig. 6B). Given its location at the rim of the central β-sheet of the GTPase domain, the 80-loop might have a role in the BSE domain movement upon GTP hydrolysis or in oligomerization. Due to its distant location from the active site, the 80-loop is unlikely to participate in the GTPase domain dimer interface. In our crystal structures, the 2-fold axis of space group P2_1_2_1_2 relates two DNM1L molecules via a polar interface of about 839 Å^2^, which resembles a typical crystal contact (Fig. 6C). Various residues of the BSE (amino acids 2, 9, 10) and the 80-loop (amino acids 72, 73, 75, 76, 86) form altogether 14 hydrogen bonds and salt bridges, involving up to 20 water molecules. Nevertheless, we cannot exclude that this novel interface in the dynamin superfamily serves as additional element for the assembly of higher DNM1L oligomers, since its architecture would allow further GTPase-GTPase domain interaction, while the two BSEs could be connected to opposing stalk filaments. Furthermore, small interfaces mediating low affinity interactions are often found in membrane-bound oligomeric complexes [55].

While the overall negatively charged 80-loop may interact with positively charged regions of other protein molecules, some of the seven glutamates and aspartates could serve as cation binding ligands for Mg^2+^ or even Ca^2+^, which sometimes reaches micromolar levels at mitochondrial membranes [56], [57]. Intriguingly, the 80-loop sequence resembles those of Ca^2+^-binding proteins, such as serine proteases and phospholipid binding proteins [58], [59].

### GTPase Activity Determinants

**Figure 7:**
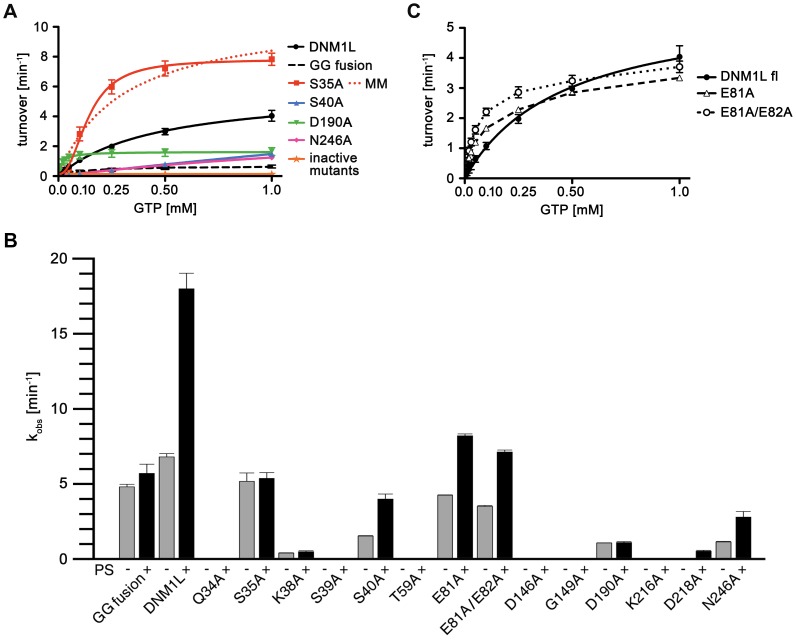
GTPase activity of DNM1L and the mutants. (**A**) Basal GTPase activities of wild-type DNM1L, DNM1L GG fusion protein and full-length mutants. Steady-state GTPase activities of full-length wild-type DNM1L, GG fusion protein, active site mutants and predicted GTPase domain dimerization mutants (Q34A, S35A, D190A) were measured as described in the Methods section. Amino acid substitutions Q34A, K38A, S39A, T59A, D146A, G149A, K216A and D218A completely abolished GTP hydrolysis. The Q34A mutant is shown as one representative example for the inactive mutants. Among all these mutants, only S35A, S40A, D190A and N246A exhibited significant GTPase turnover. For S35A, both the simple Michaelis-Menten equation fit (label MM, orange dots) and the curve using a cooperative model (continuous orange line) with a Hill coefficient of 2.2 are depicted. Data are means of at least three independent experiments ± standard deviation (displayed as error bars) evaluated by nonlinear regression analysis. (**B**) Liposome-stimulated GTP hydrolysis of DNM1L and its mutants determined by multiple-turnover assays. Reactions were performed for 12 min at 37°C in the absence (grey bars) or presence (black bars) of PS liposomes. Initial hydrolysis rates k_obs_ were determined by applying a linear fit to the data, with bars representing mean value ± standard deviation of three independent experiments. For mutants Q34A, S39A, T59A, D146A, G149A, and K216A less then 4% of the GTP was hydrolyzed within 12 minutes. (**C**) Basal GTP activity of full length DNM1L and the two loop mutants E81A and E81A/E82A. Although the three variants exhibit similar Michaelis-Menten curves, both mutants displayed lower V_max_ (k_obs_) and faster saturation with GTP compared to WT.

Based on the DNM1L GG fusion structure, a systematic analysis of critical residues for GTP binding and turnover was undertaken using full-length DNM1L mutants. The enzyme-coupled assay with increasing GTP concentration resulted in characteristic saturation curves for the GTPase reaction of 1.2 µM DNM1L, resembling Michaelis-Menten kinetics (Fig. 7A, Table 3). The apparent maximal GTPase rate was about 6 min^-1^, with a GTP concentration for half-activation of about 460 µM. However, Michaelis-Menten kinetics assumes a simple enzyme-substrate reaction and does not take into account the underlying multi-step GTPase reaction mechanism of dynamin superfamily proteins [8]. Thus, increasing nucleotide or protein concentrations might induce GTP-dependent interactions of the GTPase domains resulting in higher apparent reaction rates.

**Table 3:** Kinetic parameters of DNM1L basal GTPase activities.

DNM1L	k_obs_ (min^−1^)^a^	K (µM)^a^	k_cat_/K_m_ (M^−1^ min^−1^)	relative k_obs_ b	Motif	Function
WT	5.84±0.18	462±30	12630±420	100		
GG fusion	0.69±0.05	120±26	5740±160	12		
Q34A	0.0	–	–	0	G1 (P-loop)	γ-phosphate binding, G-dimerization
S35A	10.89±0.64	298±44	36540±1730	187	G1 (P-loop)	β, γ-phosphate binding, G-dimerization
S35A *cooperative model*	7.77±0.14	138±5	*Hill coefficient* 2.2±0.1	133		
K38A	0.0	–	–	0	G1 (P-loop)	α, β-phosphate binding
S39A	0.0	–	–	0	G1 (P-loop)	α-phosphate binding
S40A^c^	>1.0	>1000	1670±640	>20	G1 (P-loop)	α-phosphate binding, ribose via H_2_0
T59A	0.0	–	–	0	G2 (switch I)	coordination of catalytic H_2_O and Mg^2+^
E81A	3.50±0.06	102±5	34310±180	60	80-loop	unknown
E81A/E82A	3.79±0.06	69±4	54970±210	65	80-loop	unknown
D146A	0.0	–	–	0	G3 (switch II)	coordination of K38
G149A	0.0	–	–	0	G3 (switch II)	coordination of catalytic and bridging H_2_O
D190A	1.64±0.05	16±2	102190±210	28	trans-stabilizing loop	G-dimerization
K216A	0.0	–	–	0	G4	ribose binding and guanine orientation
D218A	0.0	–	–	0	G4	guanine binding
N246A^c^	>1.0	>1000	1800±790	>20	G5	guanine binding

a In all cases except for the cooperative model with the mutant S35A, k_obs_ and K correspond to k_cat_ and K_m_ of the applied Michaelis-Menten model;

b WT = 100; ^c^ k_obs_ and K could not be determined in a reliable manner, since the substrate did not reach the range of saturating levels.

To determine whether DNM1L shows liposome-stimulated GTPase activity, GTPase reactions were measured at saturating GTP concentrations (1.5 mM) and higher protein concentrations (10 µM) allowing cooperative membrane binding. DNM1L binds equally well to liposomes constituted of a typical mitochondrial membrane composition or phosphatidylserine (PS) [33], [39], [52]. Since the liposomes employed in these assays interfered with the continuous coupled reaction, an HPLC-based GTPase assay was used. Furthermore, since divalent cations induce clustering of negatively-charged liposomes, lower concentrations of Mg^2+^ (0.5 mM) were employed compared to the continuous coupled assay. Previous experiments indicated that this Mg^2+^ concentration is optimal to observe the DNM1L stimulated GTPase activity with PS liposomes [33]. Under these conditions, DNM1L showed a similar maximal GTPase rate of 7 min^−1^ that could be 2.5-fold stimulated by liposomes (Fig. 7B). This extent of GTPase stimulation is, however, much lower than the 200-fold stimulation observed in dynamin-1 under similar conditions [14].

At a concentration of 1.2 µM, the DNM1L GG fusion protein showed a reduced maximal GTPase activity of 12%, most likely due to its inability to oligomerize via the stalks [38]. At higher protein concentrations of 10 µM, the GG construct exhibited 70% of the GTPase activity of DNM1L pointing to a protein-concentration dependent increase of the GTPase (Figs. 7A, B). Addition of liposomes did not stimulate the GTPase rates, which can be attributed to the missing stalks and/or B-inserts mediating lipid binding.

Mutagenesis of single active site residues within the GTPase binding motifs G1–G5 of full-length DNM1L (Figs. 2, 4, and 5) resulted in most cases in a complete loss of GTPase activities (Fig. 7A, B, and Table 3). Thus, the P-loop mutants K38A and S39A, which are involved in phosphate binding, exhibited essentially no GTP turnover independent of the protein concentration or the presence of liposomes. A similar lack of activity has been reported before for mutant K38A [60]. Switch I residue Thr59 is involved in the positioning of the catalytic water molecule and Mg^2+^ coordination in dynamin-1 [8]. The corresponding T59A mutant in DNM1L as well as the switch II mutants D146A and G149A were also inactive (Fig. 7A, B). The D146A mutation is expected to destabilize the conformation of P-loop residue Lys38, whereas G149A might prevent binding of the catalytic water molecules. Moreover, the G4 motif mutant K216A was inactive, most likely due to a reduced coordination of the GTP ribonucleotide moiety. The P-loop mutation S40A and the G5 motif mutation N246A showed some residual GTPase activity, which could be stimulated by liposomes (Fig. 7B). However, these mutants could not be saturated with GTP pointing to a reduced affinity for this nucleotide (Fig. 7A).

To explore the function of the 80-loop, we mutated some of the negatively-charged residues and characterized its effect. The two loop mutants E81A and E81A/E82A exhibited slightly different GTP saturation curves compared to wild-type (WT) DNM1L in the continuous-coupled activity assay, such as lower V_max_ (k_cat_) and increased affinity for GTP at half-maximal turnover (K_m_) (Fig. 7C, Table 3). Also, at higher protein concentrations k_obs_ values of both mutants were significantly reduced, reaching about 62% (E81A) and 52% (E81A/E82A) of the WT DNM1L turnover (Fig. 7C). Interestingly, the GTPase activity of these mutants was enhanced by PS liposomes to only 46% of the WT activity in case of the E81A mutant and only 40% for E81A/E82A (Fig. 7B). These data might indicate a function of the 80-loop in higher-order oligomerization, or in the efficient mechanochemical energy conversion of DNM1L, perhaps mediated by the 2-fold BSE/80-loop interface.

### Determinants of GTPase Domain Dimerization Linked to Activity

**Figure 8:**
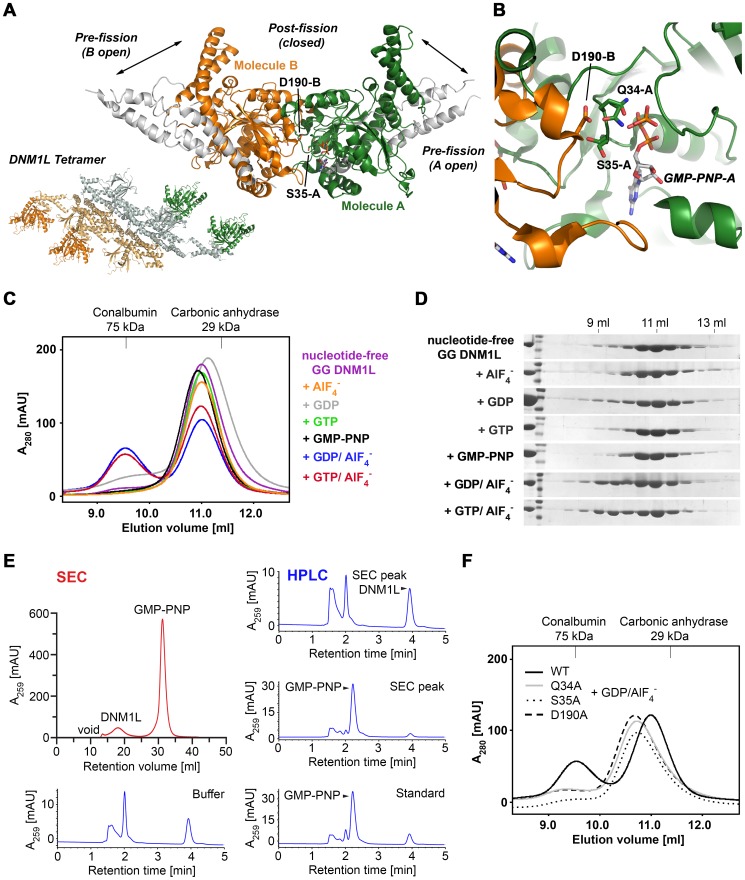
GTPase domain interface model of the DNM1L GG fusion protein and nucleotide-dependent dimerization. (**A**) Two chains of DNM1L molecules were superimposed on the GTPase domain dimer of *At*Drp1A (PDB code 3T34) as molecules A (green) and B (orange). The interface connecting residues Gln34, Ser35, Asp190, and GTP are depicted as stick models. In addition, the movement of the BSE domains between the pre- and postfission states is represented by the extended *At*Drp1A dimer (white) and the compact DNM1L dimer. The tetramer model (bottom, left) is based on full-length dynamin-1, which may further oligomerize via the stalks and other GTPase domains (green, orange). (**B**) Close-up view of the interface at Asp190 from molecule B and Gln34, Ser35 and GTP from molecule A. The conformations of the nucleotide-free and GMP-PNP bound structures are displayed. (**C**) Dimerization ability of the DNM1L GG fusion protein in the presence of different nucleotides. The GG fusion protein (60 µM) was subjected to gel filtration after incubation with different guanine nucleotide analogs (2 mM). Protein standards at 29 and 75 kDa are indicated. The dimeric protein eluted at a retention volume of 9.5 ml and monomeric protein at 11 ml. (**D**) SDS PAGE analysis of the SEC runs. Lane 1 shows purified GG fusion protein (41 kDa) followed by a molecular weight protein ladder (from top to bottom: 55 kDa, 43 kDa, 34 kDa). Elution volumes are indicated above. (**E**) Analysis of the DNM1L GMP-PNP complex stability under SEC conditions as in Fig. 8C. SEC elution (red) and further analysis of the peaks by HPLC (blue), with the indicated controls. (**F**) SEC of GG fusion protein mutants Q34A, S35A and D190A under conditions as in Fig. 8C in the presence of GDP⋅AlF_4_^−^. Retention volumes of molecular weight standards are shown above.

GTPase domain dimerization of human dynamin-1 and *Arabidopsis thaliana* dynamin-related protein A (*At*Drp1A) was shown to be mediated via a conserved interface across the nucleotide-binding site. A prominent feature of this G-dimer is a hydrogen bond network formed between an aspartic acid side chain of the trans-stabilizing loop (2X2E: Asp180, 3T35: Asp186) with a glutamine and a serine of the P-loop in the opposing molecule (2X2E: Gln40, Ser41, 3T35: Gln43, Ser44). Additionally, a serine and a glycine from switch I (2X2E: Ser61 and Gly62, 3T35: Thr64 and Gly65) form hydrogen bonds with Asp180. Overall, these interactions occur twice per G-dimer in a symmetrical manner, involving a rotational 2-fold axis [8]. All of these residues are conserved in DNM1L. Thus, we postulated that DNM1L employs a similar dimerization-dependent GTPase mechanism. Using the GDP-AlF_4_^–^bound *At*Drp1A dimer as a template, we created a model for the DNM1L dimeric GTPase domain (Fig. 8A). In this model, Asp190 in DNM1L, which corresponds to Asp180 in dynamin-1, mediates GTPase domain dimerization by binding to Gln34 and Ser35 in the P loop of the opposing catalytic site. Concomitantly, Asp190 binds to Thr55 and Gly56 from switch I (Fig. 8B).

To confirm this model, size-exclusion chromatography (SEC) experiments in the absence and presence of guanine nucleotides were performed. These measurements revealed that the DNM1L GG fusion protein is predominantly monomeric in the absence of nucleotides and upon incubation with GDP, GTP or GMP-PNP (Fig. 8C, D). In fact, GMP-PNP was efficiently separated from DNM1L over the gel filtration run pointing to a low affinity interaction of nucleotides with DNM1L (Fig. 8E). However, in the presence of GDP/AlF_4_^−^, a transition state analogue of the GTPase reaction, a significant shift to a dimeric species was observed (Fig. 8C).

In order to investigate if Gln34, Ser35 and Asp190 are involved in GTPase domain dimerization, as predicted by the dimer model, these residues were replaced by alanine residues in the DNM1L GG fusion protein. Indeed, all three mutants dimerized with reduced efficiency in comparison to the wild-type DNM1L GG fusion protein in the presence of the transition state mimic (Fig. 8F).

In both GTPase assays, the Q34A mutant did not show any GTPase activity. Since Gln34 is not only involved in dimerization, but also in nucleotide binding, this mutation might interfere with GTP binding. However, both D190A and the Ser35A mutant showed efficient GTP hydrolysis. The D190A mutant displayed even higher GTPase rates at lower nucleotide concentrations, which leveled off at high GTP concentrations. The S35A substitution led to a roughly 2-fold higher basal turnover rate at low and at high GTP concentrations. Remarkably, this mutant exhibited a distinct sigmoidal curve for the turnover of GTP, which agrees best with a cooperative model, resulting in a Hill coefficient of 2.2 (Table 3, Fig. 7A). In contrast, no significant positive cooperativity was observed for the other investigated DNM1L variants with respect to increasing substrate concentrations. Both S35A and D190A completely lost their liposome-stimulated GTPase activity (Fig. 7B). These data suggest that Ser35 and Asp190 in DNM1L are indeed involved in GTPase domain dimerization and the subsequent stimulation of the GTPase reaction.

## Conclusions

**Figure 9:**
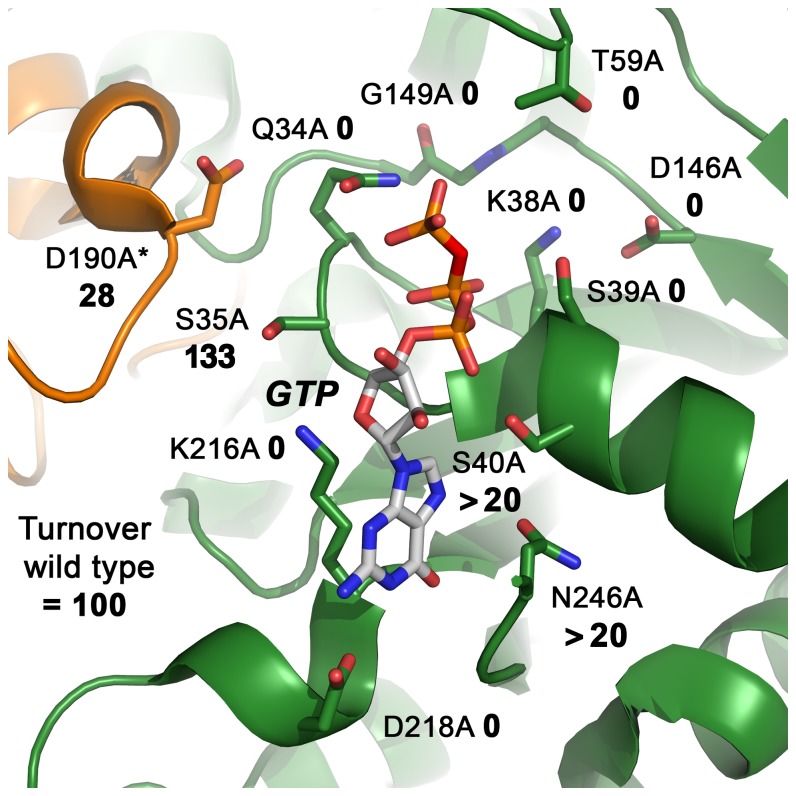
Structure-function map of the modelled DNM1L active site dimer. All active site and dimerization residues that have been mutated to alanine are represented as stick models, as well as the GTP. The turnover numbers of the respective mutants as determined by the GTPase assay for basal activity are shown, whereby the WT was defined as 100%. Molecule A of the dimer is depicted in green, while the second molecule B is shown in orange, with the corresponding D190A*.

**Figure 10:**
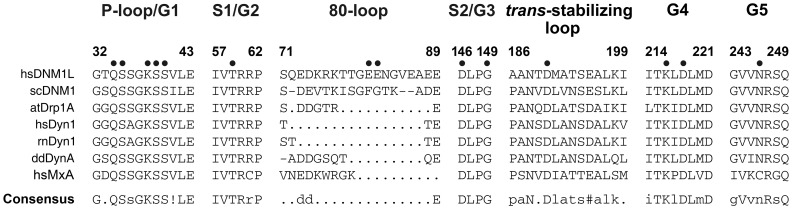
Conserved sequence motifs of the dynamin superfamily. Sequence alignments of representative members of the dynamin superfamily comparing conserved GTP binding motifs and the *trans*-stabilizing loops important for G dimerization. Conserved key residues that were mutated to alanine in our study are marked with a dot. Species abbreviations are hs, *Homo sapiens*; rn, *Rattus norvegicus*; sc, *Saccharomyces cerevisiae*; dd, *Dictyostelium discoideum*; at, *Arabidopsis thaliana*; MxA, interferon-induced GTP-binding protein A; ! represents one of the amino acids of IV; # represents one of the amino acids NDQE.

Our study provides a mutational and kinetic analysis of GTP recognizing and hydrolyzing residues in DNM1L, which were identified in the nucleotide-free and GMP-PNP crystal structures. These findings are summarized in a structure-function map of the DNM1L active site (Fig. 9). A sequence comparison of DNM1L with dynamin-1-like proteins from *S. cerevisiae* and *A. thaliana*, dynamins-1 of human, rat, and *D. discoideum*, as well as human Myxovirus resistance protein A (MxA, Mx1), highlights the significance of our investigation for understanding the common mechanisms of these mechanochemical GTPases (Fig. 10). The importance of a functional GTPase in DNM1L is emphasized by studies on cultured mammalian cells, which formed unnaturally large mitochondrial clusters or networks upon transfection with P-loop and switch I mutants of DNM1L, such as K38A and T59A [29]. An analysis of rat DNM1L mutants corresponding to K38A and D218N showed similar effects, leading to cellular lethality [61]. Similarly, the K38A DNM1L mutation affects the segregation of peroxisomes [62]. Thus, it can be expected that other GTPase defective DNM1L mutations will result in failure of mitochondria and peroxisome division. Moreover, mutations at the DNM1L G-dimer interface, such as Q34A, S35A, and D190A, might have similar effects on mitochondrial remodeling. Certainly, the formation of higher-ordered oligomers of DNM1L is crucial for its physiological function, as corroborated by the oligmerization-deficient point mutation in the stalk of human DNM1L, A395D, which is lethal for infants [63].

Future studies have to explore the fine details of mechanochemical energy conversion by full-length DNM1L with structural methods, and link the underlying mechanisms to the dynamic reality of mitochondrial and peroxisomal segregation. In particular, the roughly 100-fold lower stimulatory effect of lipids on the GTPase activity of DNM1L compared to dynamin-1 has to be elucidated. The large difference of these GTPases in the extent of their stimulated activity is not evident on the basis of the basic architecture of their GTPase domains. However, DNM1L oligomerization at the mitochondrial surface and stronger GTPase stimulation might depend on specific membrane receptors such as MFF, which was not present in our assays [34]. Moreover, the lower GTPase activation of DNM1L might be based on the different oligomerization modes via the stalks, which have been proposed for dynamin and DNM1L [32], [33]. DNM1L is implicated in several neuronal diseases, such as Alzheimer’s, Parkinson’s, Huntington’s, and amyotrophic lateral sclerosis [64]. Eventually, the structure-based knowledge of regulating the function of DNM1L by pharmacological means may help to target these neurodegenerative diseases successfully.
